# Fuzzy Logic Analysis for Key Factors for Customer Loyalty in E-Shopping Environment

**DOI:** 10.3389/fpsyg.2021.742699

**Published:** 2021-09-29

**Authors:** Li-Xin Guo, Chih-Chen Lin, Po-Fang Huang, Shou-Yueh Jhou, Shih-Chih Chen, Fu-Sheng Tsai

**Affiliations:** ^1^School of Business, Huaiyin Institute of Technology, Huai'an, China; ^2^Postgraduate Program in Management, I-Shou University, Kaohsiung, Taiwan; ^3^Physical Office, Fu Jen Catholic University, New Taipei City, Taiwan; ^4^China Life Insurance Co., Ltd., Taipei, Taiwan; ^5^Department of Information Management, National Kaohsiung University of Science and Technology, Kaohsiung, Taiwan; ^6^Department of Business Administration, Cheng Shiu University, Kaohsiung, Taiwan; ^7^Center for Environmental Toxin and Emerging-Contaminant Research, Cheng Shiu University, Kaohsiung, Taiwan; ^8^Super Micro Mass Research and Technology Center, Cheng Shiu University, Kaohsiung, Taiwan

**Keywords:** fuzzy logic, loyalty, e-shopping, digital economy, consumer

## Abstract

Due to subjective evaluation and qualitative characteristics, the assessment processes of loyalty often cannot use the crisp value to express the final value. That makes the evaluation of online store performance usually filled with uncertainty and ambiguity. The loyalty assessment of electronic shopping is complex, and online stores' strategy and production control problems are frequently accompanied by uncertain conditions. This study constructs a conceptual model to leverage the fuzzy logic approach for understanding the consumers' decision-making in the e-service context of shopping. This study also exposes the relationships between system quality, information quality, and service quality to understand their impacts on customer loyalty. Implications and future research directions for service providers and researchers are discussed.

## Introduction

The growing popularity of the Internet and information technology continues to flourish. In such a background, the use of mobile (web) sites are growing every year. According to the latest information on Internet World Stats, as of June 2008, the global online population has reached 1.46 billion, of which Asia accounted for 40%, 26% in Europe, and 17% in North America. Penetration of the global Internet population has surpassed into two, which in North America up to 74%, 60% in Oceania, and 48% in Europe. Global Internet population from 2000 to 2008 grew at a rate of more than 300%, and the countries of the world strive to improve the information penetration of the case. Therefore, the global Internet population will show a more rapid population growth in the future^1^. In addition, in investigation results from International Telecommunication Union, twice the world's online population has grown over the past 5 years, and will include more than 20 million people this year^2^. Then according to the Institute for Information Industry statistics, at the end of June 2010 in Taiwan, total broadband subscribers were 4.98 million, an increase over the previous quarter's fifty thousand users^3^. The global Internet used by the rapid rise in population could reflect how network usage and daily life have been inextricably linked.

With the rapid development of Internet, along with the number of people e-shopping has continued to grow in recent years. According to a Market Intelligence Center study, global e-shopping market grew from in 2006 at U.S. 640 billion dollars to U.S. 782 billion dollars in 2008. And the forecasts for 2009 and 2010, respectively, are up to U.S. 830 billion dollars and U.S. 951 billion dollars. Although the development of Taiwan's e-shopping is late, after a strong government and private investment in information and communication building, the Internet use of the environment has matured, and Taiwan's e-shopping market will reach U.S. 105 billion dollars in 2009^4^.

Compared to physical stores, online stores possess lower spatial and temporal costs, low barriers to construct, and are coupled with media reports. Therefore, attracting many people into the online store to entrepreneurship, making the online store has become the most popular emerging industry. But also because of the relatively low barriers to entry, online store competition is fierce; therefore, to enhance the performance of online stores becomes even more important, and the online store's performance depends on many factors. For example, the characteristics of goods, the smoothness of the website, the way of transactions, distribution, and other factors (Troy and Shaw, 1997) will affect the online store crowds and the money flows, and also the online store's performance in the operating performance. Since the influencing factors are complex, it is difficult to control the online store's business strategy. In fact, for such decision problems with uncertainty and ambiguity, we should be able to use fuzzy set theory to deal effectively and make the appropriate decisions (Bellman and Zadeh, 1970; Chen and Hwang, 1992; Akhter et al., 2003).

In this study, we use the linguistic variables to express the subjective assessment value of the evaluators in order to reduce the ambiguity. We then use the combination of fuzzy logic theory and neural algorithm to assess a proper decision to construct a decision model for the performance of the online store. In this way, we can quickly and effectively grasp the operation of the online store for the consumers and enterprises through the online store performance of decision-making. Based on the above, the main purposes of this study are the following two points:

(1) Discussion the performance influencing factors of online store.
(2) Construct a fuzzy logic for shopping online store management decision-making.

## Literature Review

### E-Commerce and E-Shopping

Due to the Rapid progress of Internet technology, e-commerce's management become an important research topic. E-commerce is a kind of way of business communication and transaction by computer and Internet (Haynes, 1995). Broadly speaking the exchange of the commercial activity can be called e-commerce (Wigand, 1997). Turban et al. (1999) also defined e-commerce through the Internet to sell or exchange products, services, and information. This shows that the e-commerce utilizes the Internet for commodities trading.

Online stores can be called electronics stores or online market. The main feature of online stores is consumers use the Internet to browse and purchase goods. But the online store is a virtual store; consumers only rely on photos, images, and product description which the Web site provides to know the status of goods. Therefore, the transaction process has considerable risk. And how to maintain good relations with buyers and sellers is a very important management mechanism of the online store's manager.

### Information System Success

**Figure 1:**
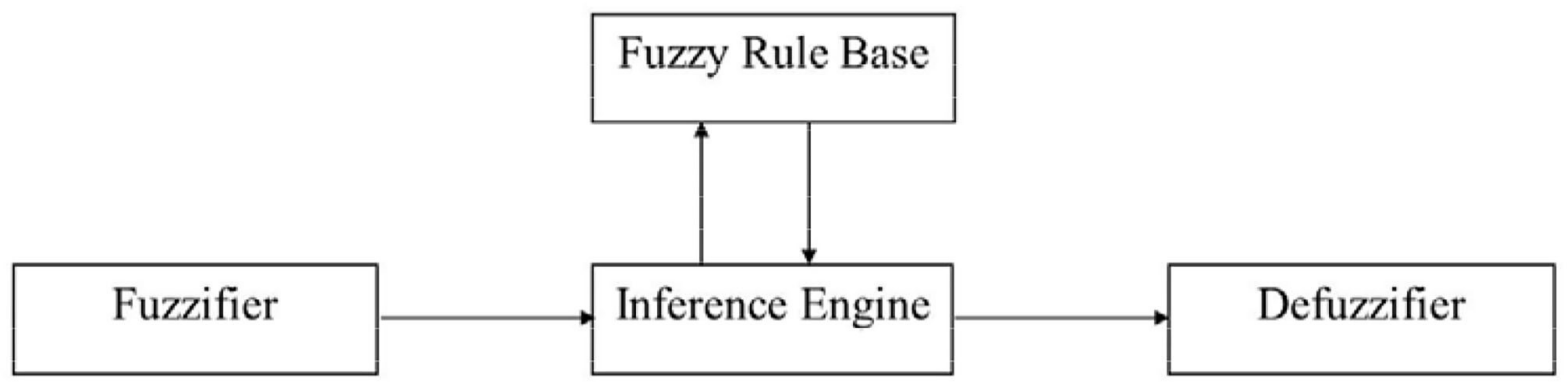
Block diagram of fuzzy logic controller.

Many literatures have studied the influencing factors of e-shopper's loyalty, for example, Tankovic and Benazic have analyzed how e-servicescapes affect perceived e-shopping value and ultimately influence e-shopper's attitude loyalty, and they find that the dimensions (such as layout, functionality, and financial security) of e-servicescape influence and determine the perceived e-shopping value point (Tankovic and Benazic, 2018). Cachero-Martínez and Vázquez-Casielles have tested how the e-shopping experience impacts the customers' attitudinal and behavioral loyalty through emotional experience (Cachero-Martínez and Vázquez-Casielles, 2021). Bhaskar and Kumar theorize and identify three main factors: service quality, customer satisfaction, and trust as positively related to the customers' loyalty in e-commerce based on the satisfaction-trust-loyalty theory (Bhaskar and Kumar, 2016). Al-Khayyal et al. have examined the influence of e-service quality (including website design, privacy, security, efficiency, and customer service/communication) on e-shoppers' loyalty (Al-Khayyal et al., 2020). Shafiee and Bazargan have investigated by questionnaire and found that e-service quality (which is directly influenced by information security and website performance) has a positive impact on e-loyalty through its positive effects on e-recovery (Shafiee and Bazargan, 2018). To sum up, the existing research mainly focuses on the influence of e-shopping scenarios, e-shopping experience, e-service quality, and other factors on customers' e-loyalty by the empirical methods of statistical data analysis, and there are no studies which have integrated system, information, and service quality into a unified framework to examine its influences on customers' e-loyalty (see Figure 1).

DeLone and McLean (1992) extensively reviewed previous studies on information system success and presented an information system success model. They posit six dimensions which are system quality, information quality, use, user satisfaction, individual impact, and organizational impact to assess. The research results that the system quality and information quality are two important dimensions of the system characteristics. Both system and information quality are influential for user satisfaction.

But the DeLone and McLean model 1992 did not discuss the service quality. However, a number of scholars found the importance of service quality in information system (Kettinger and Lee, 1997; Pitt et al., 1997; Van Dyke et al., 1997). Therefore, DeLone and McLean proposed an updated information system success model in 2003 (DeLone and McLean, 2003). Use the system quality, service quality, use, user satisfaction, and net benefits to measure whether the success of information system. DeLone and McLean said that the updated model was suitable for the e-commerce environment. And more related research has confirmed the service quality which has a positive and significant impact to behavioral intention, satisfaction, and loyalty (Lai, 2004; Parasuraman et al., 2005; Yang et al., 2005; Hu et al., 2009; Kuo et al., 2009).

### Fuzzy Logic

Zadeh (1965) proposed a “Fuzzy sets” which start the beginning of the concept of fuzzy logic. It applied to define the human thinking. Thus, to solve the transitional concept “crisp set” it cannot define such as “usually” or “very far.” Fuzzy logic is a multi-valued logic focusing on developing better reasoning and decision-making models. Essentially, it is a qualitative approach for analyzing behaviors in complex systems, in which linguistic but not numerical variables are described.

**Figure 2:**
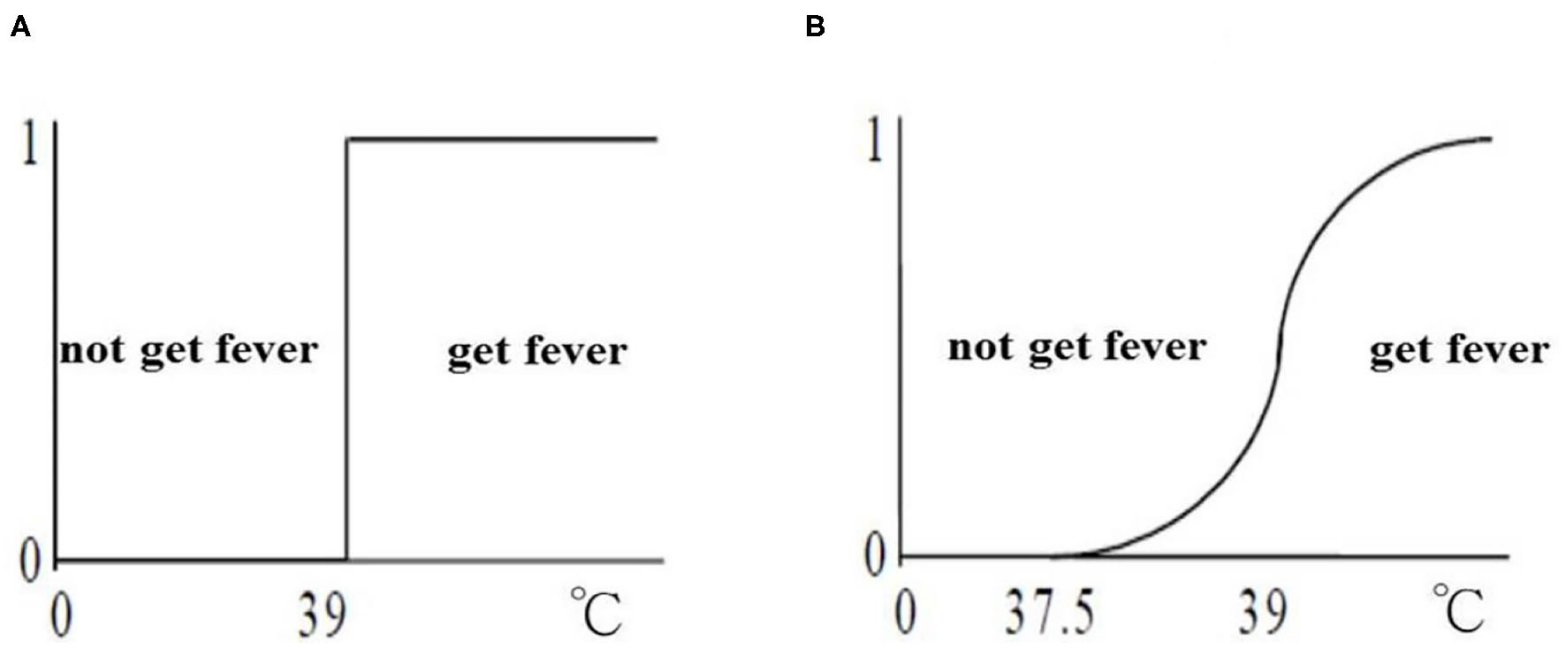
**(A)** Classical set and **(B)** fuzzy set.

Unlike traditional set theory, fuzzy set uses the concept of membership to classify elements into a continuous set. The membership function not only gives 0 or 1, but it also give values between 0 and 1. For example, a doctor agrees that the standard value is 39°C to consider something as a fever. The classical set of “get fever” is higher than 39°C. But 38.99°C is not part of a fever. However, it is not reasonable for the only difference of 0.01°C that categorize between getting fever or not. Figure 2 shows the difference between classical set and fuzzy set. In the online shopping process, users often rely on common sense and use ambiguous terminology when making purchase decisions. Considering the choice of similar alternative products and services, the online customer usually creates a certain ambiguity in his/her mind (Akhter et al., 2005; Mohanty and Bhasker, 2005; Castro-Schez et al., 2011; Sadikoglu, 2017).

Fuzzy logic controller has gained wide applications for its simplicity to apply as mathematical modeling and is not a pre-requisite. Typical fuzzy logic controller comprises four principal components: Fuzzifier, Fuzzy Rule Base, Inference Engine, and Defuzzifier.

The Fuzzy Rule Base keeps record of the process operation containing “if-then” rules. The Inference Engine is the core of a Fuzzy logic controller. Based on approximate reasoning, the controller has the capability of simulating human decision making. During the process, it derives a reasonable action for specific situation based on the given rule base. Lastly, the Defuzzifier converts the fuzzy control action to the non-fuzzy action that fits the real world.

## Structure and Procedures

**Figure 3:**
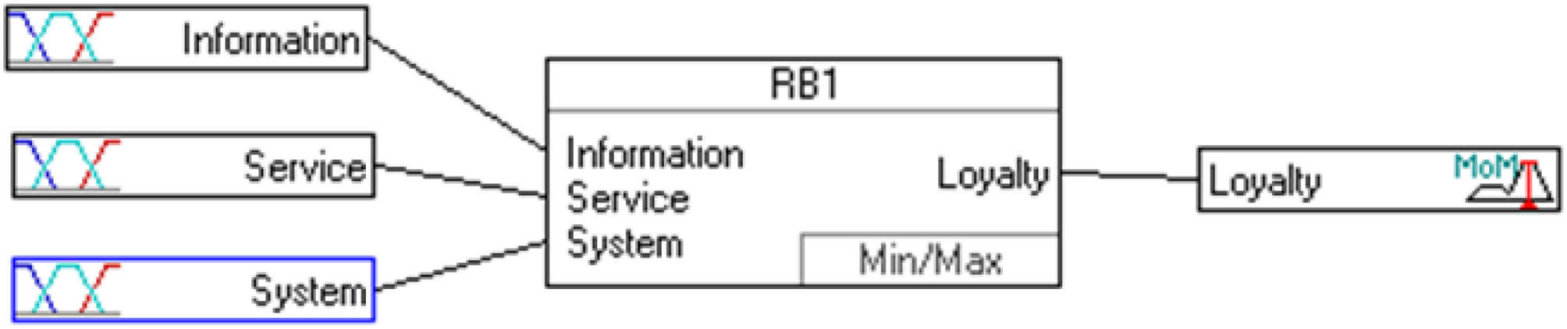
The proposed framework.

**Figure 4:**
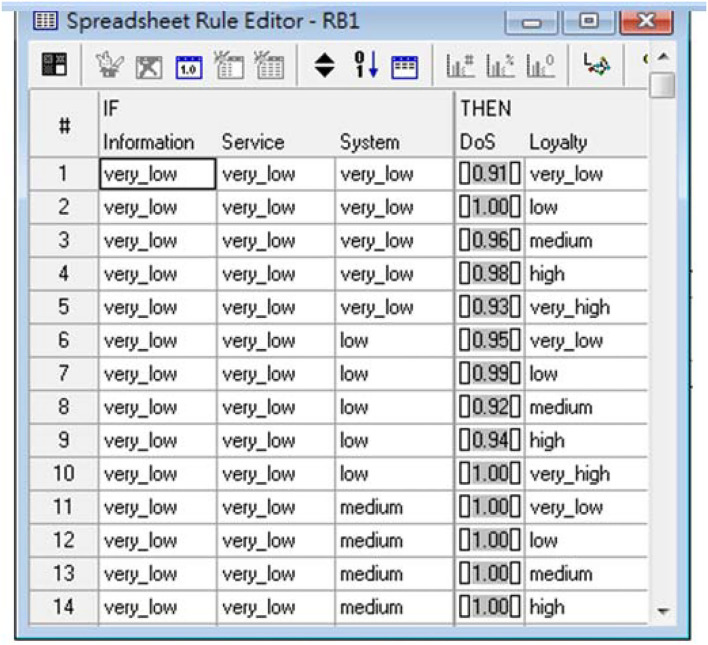
Partial decision rules for measuring the possibility of maintenance.

**Figure 5:**
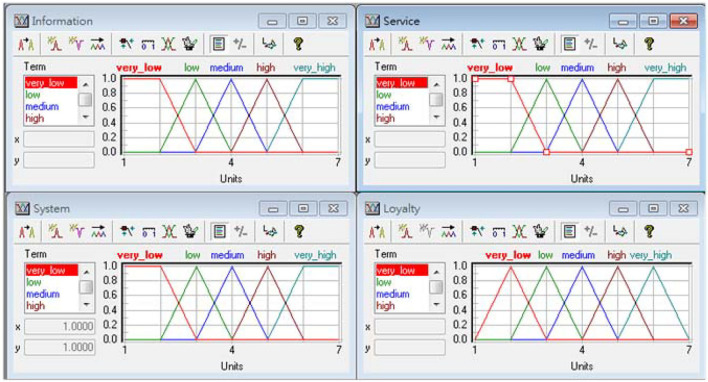
Member functions.

In recent years, because of the rapid development of Internet applications and Information Technology, online shopping adopted information system use to create a competitive advantage. In this study, DeLone and McLean (2003) proposed information system success model. They measure the loyalty of the e-shopping, whether it has significant impact on the information quality, service quality, and system quality. This study takes the fuzzy with three inputs and one output as an example. The architecture of fuzzy is shown in Figure 3. Table 1 presents the respective linguistic members of the summarized variables. Decision rules can usually be represented in the form “If … Then …” Figure 4 shows partial decision rules in spreadsheet format. The membership functions of respective decision variables are depicted in Figure 5.

**Table 1:** Summary of decision variables and corresponding linguistic members.

**Variables**	**Linguistic members**
System	Very-Low, low, medium, high, very-high
Information	Very-Low, low, medium, high, very-high
Service	Very-Low, low, medium, high, very-high
Loyalty	Very-Low, low, medium, high, very-high

## Results, Discussions, and Conclusion

**Figure 6:**
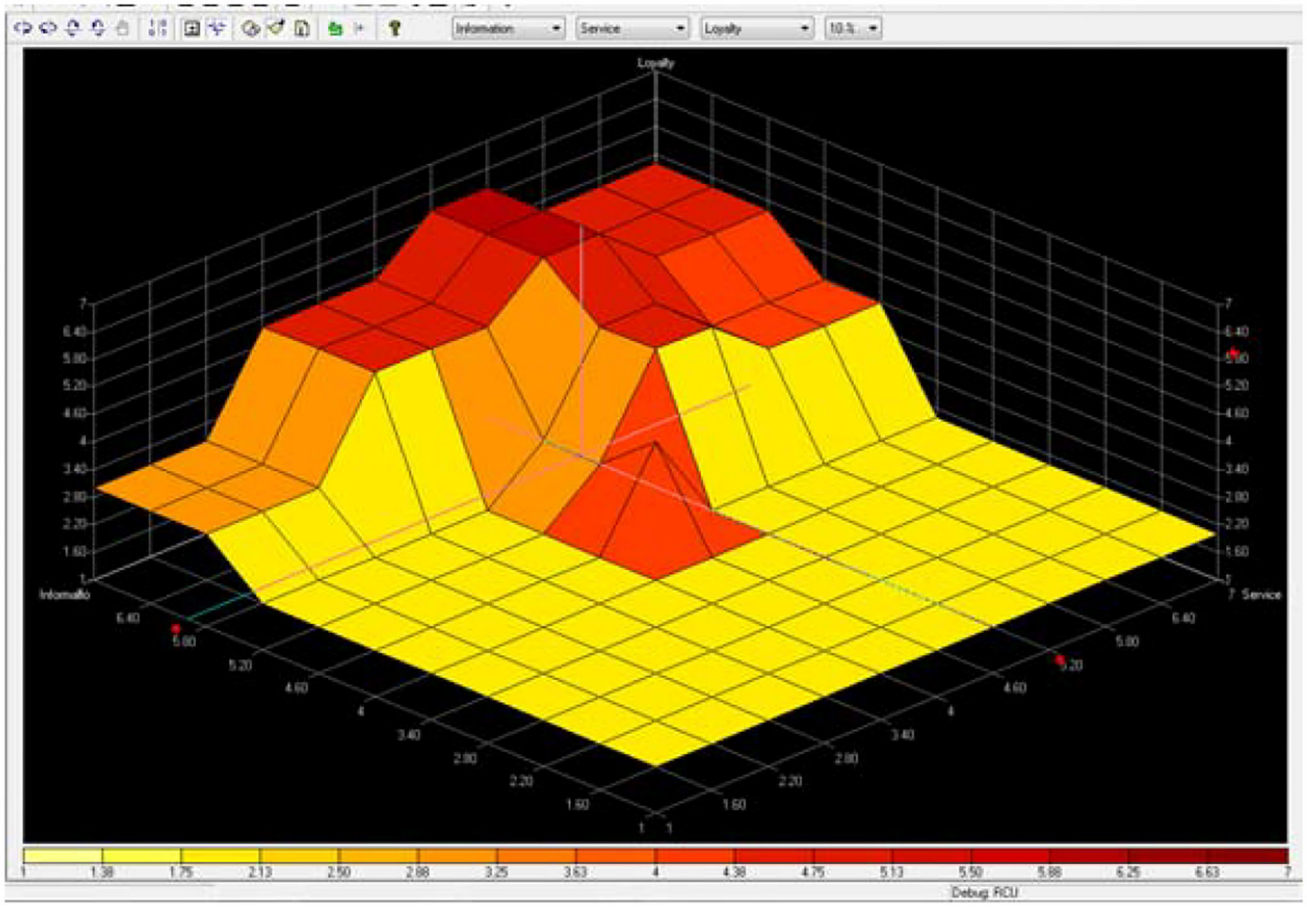
Loyalty is positively to information and service.

**Figure 7:**
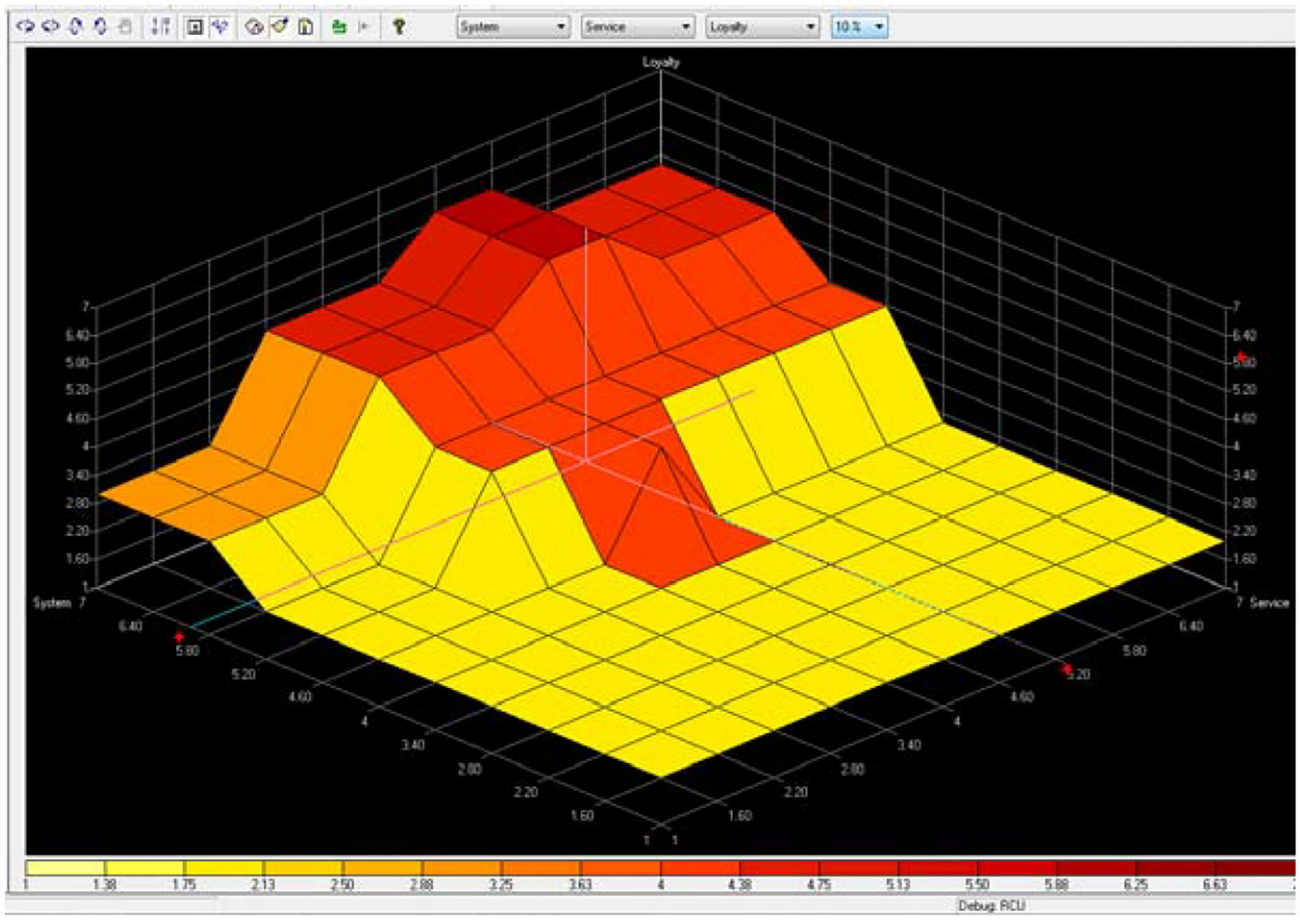
Loyalty is positively to system and service.

**Figure 8:**
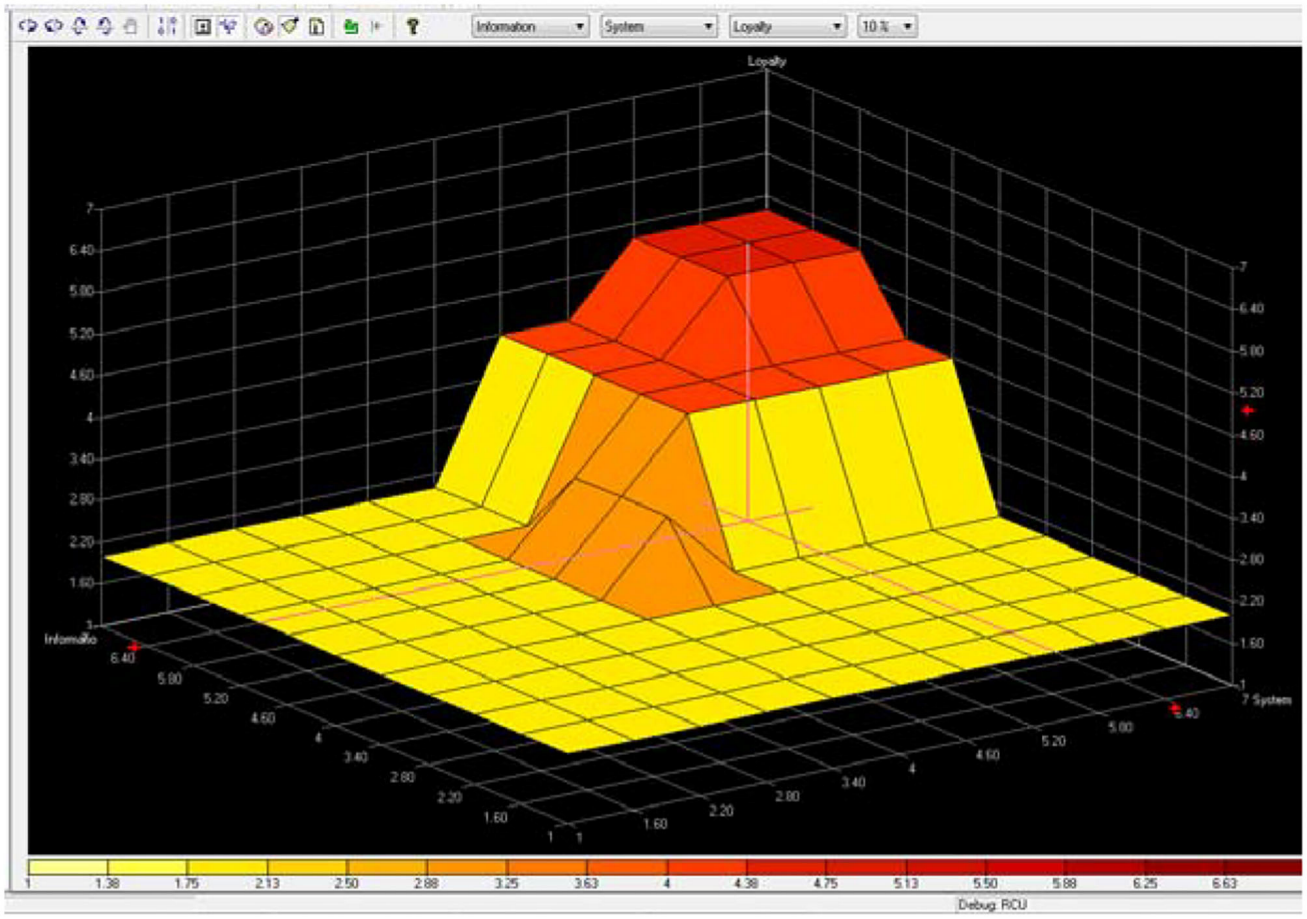
Loyalty is positively to system and information.

Compared to other e-shopping studies (Zhang, 2006; Khalifa and Liu, 2007; Zhou et al., 2007), which mainly use the complex mathematical formulas to solve the problem, this study uses the Fuzzy logic rule to represent the human mind, and more and more studies use Fuzzy logic to represent the human mind (Akhter et al., 2005; Lu and Sy, 2009; Liu, 2010). Increasing choices for consumers is obvious in the context of online shopping with diverse products. In this study, the vendor would benefit from the survey data aggregated over time to refine existing rule-sets. The vendor can also utilize the data to ascertain the loyalty of the site as per user's perception and rectify if needed. Figure 6 presents that the highest gradient for loyalty is when information is “very-high” and service is “medium” to “high.” This suggests that when consumers somewhat have information with online shopping, then a small increase in service from medium to high will boost their loyalty in a significant way. Figure 7 presents that the highest gradient for loyalty is when system is “very-high” and service is “medium” to “high.” This suggests that the system quality for loyalty is influenced. So to simplify, an easy to operate system for consumers is a very important factor. Figure 8 presents that the highest gradient for loyalty is when system is “very-high” and information is “very-high.” This suggests that consumers have high quality for system and information. Easy to use and with good information quality is a very important factor for e-commerce.

## Theoretical and Practical Implications

Many existing studies have found that the system, information, and service quality of online commerce has a significant positive impact on customers' continuous use behavior (DeLone and McLean, 1992, 2003; Van Dyke et al., 1997; Tankovic and Benazic, 2018; Cachero-Martínez and Vázquez-Casielles, 2021), however, does the highest system/information/service online quality necessarily lead to the highest customer loyalty behavior? Our study results show that we cannot make a simple affirmative answer to this. From Figures 6, 7, we can find that when the quality of online service is medium to high, and the quality of information and system is very high, which leads to the highest customer loyalty, thus we believe that the relationship between system/information/service quality and loyalty is non-linear and uncertain, nor is it that the higher the quality of online services, the higher the loyalty, and there may exist the best combination of system-information-service quality that has the best impact on online customer loyalty. Therefore, our study is a further improvement and supplement to the related theoretical research on the influencing factors for customer loyalty in e-shopping.

In addition, our study also has important practical applications. First, vendors can use survey data to build a multi-dimensional graph of the relationship between system-information-service quality and customer loyalty behavior. Second, vendors can analyze and determine the spatial position of their system/information/service quality in the multi-dimensional relationship diagram. Finally, based on the spatial position, vendors can make the best decisions to increase customer loyalty. For example, with limited resources and capabilities, if the vendor's service quality is below the medium level and the information/system quality is below the high level, then the vendor can choose the method that has the least investment but the biggest effect on improving customer loyalty. For another example, if the vendor's service quality is very high, but the system/information quality is below very high, the vendor can appropriately reduce the service quality investment and increase the system/information quality investment, which can improve customer loyalty.

## Data Availability Statement

The original contributions presented in the study are included in the article/supplementary material, further inquiries can be directed to the corresponding author/s.

## Author Contributions

L-XG conceived and designed the research and provided guidance throughout the entire research process and made main revisions to the original manuscript during the interactive process of submission. C-CL and P-FH wrote and supplemented the English paper. S-YJ participated in data processing. S-CC and F-ST reviewed and edited the paper and are responsible for all R&R works. All authors contributed to the article and approved the submitted version.

## Funding

This research was partially funded by the Jiangsu Province Social Science Foundation Project, grant number: 17SHB006 (Research results of Jiangsu Social Science Fund Project, http://jspopss.jschina.com.cn/).

## Conflict of Interest

S-YJ is employed by China Life Insurance Co., Ltd., Taiwan. He joined the research when he was a graduate student in a master's program. The remaining authors declare that the research was conducted in the absence of any commercial or financial relationships that could be construed as a potential conflict of interest.

## Publisher's Note

All claims expressed in this article are solely those of the authors and do not necessarily represent those of their affiliated organizations, or those of the publisher, the editors and the reviewers. Any product that may be evaluated in this article, or claim that may be made by its manufacturer, is not guaranteed or endorsed by the publisher.

## Footnotes

^1^Available online at: http://www.find.org.tw/find/home.aspx?page=news&id=5340.

^2^Available online at: http://www.ithome.com.tw/itadm/article.php?c=64008.

^3^Available online at: http://www.find.org.tw/find/home.aspx?page=many&id=159.

^4^Available online at: http://www.cepd.gov.tw/m1.aspx?sNo=0012144.
